# Prevalence of HIV-associated esophageal candidiasis in sub-Saharan Africa: a systematic review and meta-analysis

**DOI:** 10.1186/s41182-020-00268-x

**Published:** 2020-09-23

**Authors:** Ronald Olum, Joseph Baruch Baluku, Ronald Okidi, Irene Andia-Biraro, Felix Bongomin

**Affiliations:** 1 School of Medicine, College of Health Sciences, Makerere University, Kampala, Uganda; 2 Directorate of Programs, Mildmay Uganda, Wakiso, Uganda; 3 Division of Pulmonology, Mulago National Referral Hospital, Kampala, Uganda; 4 Department of General Surgery, St. Mary’s Hospital – Lacor, Gulu, Uganda; 5 Department of Medicine, School of Medicine, College of Health Sciences, Makerere University, Kampala, Uganda; 6 Medical Research Council/Uganda Virus Research Institute and London School of Hygiene and Tropical Medicine Uganda Research Unit, Entebbe, Uganda; 7 Department of Medical Microbiology and Immunology, Faculty of Medicine, Gulu University, Gulu, Uganda

**Keywords:** Esophageal candidiasis, Prevalence, HIV/AIDS, Sub-Saharan Africa, Review, Meta-analysis

## Abstract

**Background:**

Esophageal candidiasis (OC) is a common AIDS-defining opportunistic infection. Antiretroviral therapy (ART) reduces the occurrence of OC and other opportunistic infections among persons living with HIV (PLHIV). We sought to determine and compare the prevalence of OC in the ART and pre-ART era among PLHIV in sub-Saharan Africa (SSA).

**Methods:**

We searched PubMed, Embase, Web of Science, and the African Journals Online databases to select studies in English and French reporting the prevalence of HIV-associated OC in SSA from January 1980 to June 2020. Reviews, single-case reports, and case series reporting < 10 patients were excluded. A random-effect cumulative meta-analysis was performed using STATA 16.0, and trend analysis performed using GraphPad Prism 8.0.

**Results:**

Thirteen eligible studies from 9 SSA countries including a total of 113,272 patients were qualitatively synthesized, and 9 studies were included in the meta-analysis. Overall pooled prevalence of HIV-associated OC was 12% (95% confidence interval (CI): 8 to 15%, *I*^2^ = 98.61%, *p* <. 001). The prevalence was higher in the pre-ART era compared to the ART era, but not to statistical significance (34.1% vs. 8.7%, *p* = 0.095). In those diagnosed by endoscopy, the prevalence was higher compared to patients diagnosed by non-endoscopic approaches, but not to statistical significance (35.1% vs. 8.4%, *p* = .071). The prevalence of OC significantly decreased over the study period (24 to 16%, *p* < .025).

**Conclusion:**

The prevalence of OC among PLHIV in the ART era in SSA is decreasing. However, OC remains a common problem. Active endoscopic surveillance of symptomatic patients and further empirical studies into the microbiology, optimal antifungal treatment, and impact of OC on quality of life of PLHIV in SSA are recommended.

## Introduction

Sub-Saharan Africa (SSA) remains the region most affected by the human immune deficiency virus (HIV)/acquired immune deficiency syndrome (AIDS) pandemic [1]. Of the estimated nearly 38 million people living with HIV (PLHIV) globally at the end of 2018, over 60% (~ 25.7 million) are found in SSA alone [1, 2]. In 2018, a total of 800,000 new infections and 310,0000 deaths were reported from eastern and southern Africa alone, accounting for 47% and 40% of the global AIDS-related deaths [2].

Mucocutaneous manifestations due to opportunistic infections and malignancies are the most common and usually the first recognizable clinical presentation in patients with HIV/AIDS [3, 4]. Persistent oropharyngeal candidiasis and Esophageal candidiasis (OC) or candidiasis of the trachea, bronchi, or lungs are common AIDS-defining lesions seen in PLHIV in World Health Organization (WHO) clinical stages III and IV or CD4 T lymphocyte cell count < 200 cells/mm^3^ [5]. OC typically occurs at relatively lower CD4 cell counts than oropharyngeal candidiasis [6]. High HIV viral loads have also been associated with OC independent of CD4 counts [7].

OC is the most common cause of infectious esophagitis in patients with advanced HIV disease, mainly caused by the yeast *Candida albicans* but also other non-*albicans Candida* species such as *C. glabrata, C. tropicalis, and C. dubliniensis* [8]. OC is associated with substantial morbidity and adversely affects the quality of life of the affected persons owing to the dysphagia, odynophagia, retrosternal chest pain, and extreme weight loss attributed to the disease [9]. On endoscopic examination, white mucosal plaque-like lesions, erythema, and exudates adherent to the mucosa are frequently observed [9]. Biopsy or mucosal brushing samples reveals pseudohyphae of the *Candida* species and is the preferred method of diagnosis of OC [9]. Microbiological confirmation, speciation, and antifungal susceptibility are achieved through a culture of the endoscopically acquired samples.

Between 10 and 15% of PLHIV will develop esophageal candidiasis at one point in their life-time [10]. OC is observed in over 20% of PLHIV with CD4 counts of 200 cells or less who are currently not on antiretroviral therapy (ART) [11]. Early in the HIV/AIDS epidemic in SSA, OC frequently with oropharyngeal candidiasis was observed in over 80% of hospitalized PLHIV [12]. However, with the global rollout of ART and the recent “test-and-treat strategy,” the prevalence of OC has greatly reduced due to early ART initiation [13].

We hypothesized that with over 10 years of the widespread use of ART and enhanced anti-infective prophylaxis in SSA, the occurrence of opportunistic diseases including OC is expected to have significantly reduced. However, there is a scarcity of studies to demonstrate the trend of OC in the pre-ART and ART era in SSA. Such information is not only valuable for epidemiological purposes but also help guide targeted clinical care of this vulnerable population. The aim of this study, therefore, was to determine the prevalence and the trend in the prevalence of OC among PLHIV in SSA over the past nearly 40 years of the HIV/AIDS pandemic.

## Methods

### Study design

This systematic review and meta-analysis were conducted according to the criteria outlined in the Preferred Reporting Items for Systematic Reviews and Meta-Analyses (PRISMA) statement [14]. The PRISMA checklist is available as a Supplementary File. We did not register the study protocol prior to commencement of the research.

### Search strategy

With the help of a qualified medical librarian, all studies published from January 1980 to June 2020 were searched from EMBASE, PubMed, Web of Science, and the African Journals Online (AJOL).

The following search terms were used: “oesophageal candidiasis” OR “esophageal candidiasis” OR “candidal oesophagitis” OR “candidal esophagitis” OR “*Candida* infection of the oesophagus” OR “monilial oesophagitis” AND “human immune deficiency virus” OR “HIV” OR “acquired immune deficiency syndrome” OR “AIDS” AND “Africa South of Sahara” OR “sub-Saharan Africa” OR “Angola” OR “Benin” OR “Botswana” OR “Burkina Faso” OR “Burundi” OR “Cameroon” OR “Cape Verde” OR “Central African Republic” OR “Chad” OR “Comoros” OR “Democratic Republic of Congo” OR “Cote d'Ivoire” OR “Eritrea” OR “Eswatini” OR “Ethiopia” OR “Gabon” OR “Gambia” OR “Ghana” OR “Guinea” OR “Guinea-Bissau” OR “Kenya” OR “Lesotho” OR “Liberia” OR “Madagascar” OR “Malawi” OR “Mali” OR “Mauritania” OR “Mauritius” OR “Mozambique” OR “Namibia” OR “Niger” OR “Nigeria” OR “Rwanda” OR “Sao Tome and Principe” OR “Senegal” OR “Seychelles” OR “Sierra Leone” OR “Somalia” OR “South Africa” OR “South Sudan” OR “Sudan” OR “Tanzania” OR “Togo” OR “Uganda” OR “Zaire” OR “Zambia” OR “Zimbabwe”.

Besides, we performed a manual literature search for all the references of the included articles to find applicable studies. The search was limited to human studies only. Studies written in English and French, which are the most common languages, published in sub-Saharan Africa were selected.

### Study selection criteria

Duplicate studies were identified and merged using the HDAS program. Titles and abstracts were primarily screened by two independent reviewers (F.B and R.O) to exclude those not related to this study. After the initial screening, full texts of potentially eligible studies were retrieved and examined for eligibility.

Studies that met the following inclusion criteria were included:

i. Retrospective or prospective observational studies from January 1980 to June 2020.
ii. Published in the English and French languages.
iii. Reporting prevalence of HIV-associated OC in countries located in SSA.

Studies reporting oropharyngeal candidiasis or did not specify the location of upper gastrointestinal candidiasis were excluded. Single-case reports and case series reporting less than 10 cases were also excluded from the study.

### Data extraction

A pre-designed macros form in Microsoft Excel 2016 was used to extract data from each article. The following items were extracted:

- *Study characteristics*; authors, year of publication, study country, study design.
- *Study characteristics;* age (mean or median), sex, the sample size of the participants, number of patients diagnosed with OC (prevalence), how the diagnosis was made (non-endoscopic vs. endoscopy), and the period of data collection in each study.

Two independent reviewers (RO and FB) extracted data, and any differences were resolved by discussion and consensus. The corresponding authors for eligible studies with missing data were contacted by email for clarification.

#### Operational definition

The pre-ART era was defined as patients diagnosed with HIV-associated OC before 2004 (1981–2003) and ART era between 2004 and to date. ART era was further sub-divided into early ART era (2004–2014) when ART was limited to only severely ill patients (WHO clinical stages III and IV or CD4 count of 200 cells/μl or less) [15] and late ART era (2014 to date) when ART is more available with the adoption of “test and treat” strategy for all patients diagnosed with HIV [16].

### Qualitative assessment

All the eligible studies included were assessed for risk of bias using the Critical Appraisal Skills Programme (CASP) checklist. The risk of bias in individual studies was graded as low, moderate, and high. Studies with a high risk of bias were excluded from the meta-analysis.

### Study outcomes

The study outcome is the pooled prevalence and the trend in the prevalence of OC in patients with HIV/AIDS living in SSA.

### Analysis

All analyses were performed using Microsoft Office 2016 (Microsoft Inc., Washington, USA), GraphPad Prism 8.0, and STATA 16.0 statistical software (Stata Corp, College Station, Texas, USA). A random effect cumulative meta-analysis was performed using in STATA 16.0 to illustrate the trends in the prevalence of HIV-associated OC and presented in a forest plot. Heterogeneity across studies was assessed using Q statistics and *I*^*2*^ value. A funnel plot was generated to assess for publication and sensitivity analysis performed. Mann-Whitney test was performed in GraphPad Prism 8.0. *p* < 0.05 was considered statistically significant at the 95% confidence interval (CI). Descriptive statistics were used to summarize data from individual studies. A narrative synthesis was also employed to present the results and discussion of the different studies included.

## Results

### Study characteristics

**Fig. 1:**
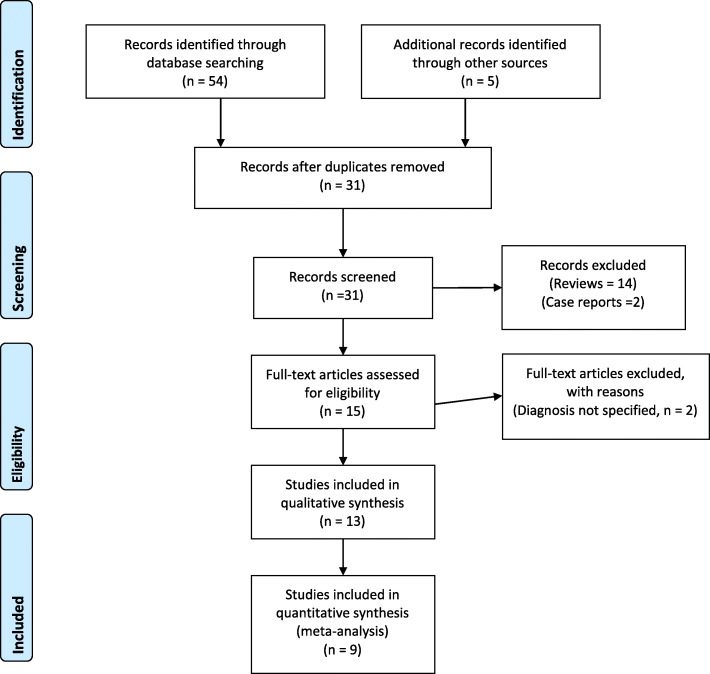
PRISMA flow diagram

Table 1 summarizes the characteristics of all the studies included in the systematic review. Thirteen studies from 9 SSA countries (Tanzania, *n* = 1; Togo, *n* = 2; Cameroon, *n* = 1; Malawi, *n* = 2; Mozambique, *n* = 1; Zambia, *n* = 1; Nigeria, *n* = 1; Kenya, *n* = 1; and Uganda, *n* = 3;) were eligible from the studies identified through database and manual literature search (Fig. 1). Only 9 studies [10, 17–24] reporting OC in general HIV populations were included in the quantitative synthesis and 4 studies [25–28] in specific HIV populations were excluded from the meta-analyses. Overall, 9,150 patients were diagnosed with OC of the 113,272 HIV patients in SSA who were assessed between 1989 and 2014. The majority of the studies (*n* = 7) were retrospective studies, and one was a randomized clinical trial. Five of the studies were conducted before the introduction of ART.

**Table 1:** Characteristics of included studies

Study	Country	Study type	Study population	Age: median or mean (IQR) years	Sex (M/F)	Patients (*N*)	Cases	Diagnosis	ART period
Mushi et al. [25]*	Tanzania	Retrospective observational	Patients attending the endoscopy unit between August 19, 2009, and September 19, 2014	–	−/−	15	8	Endoscopic	ART Era
Lawson-Ananissoh et al. [17]	Togo	Retrospective observational	Lome´ University Hospital Hepato-Gastroenterology Department records covering a 10-year period; 2005 to 2014	41	201/231	432	32	Not specified	ART Era
Andoulo et al. [27]*	Cameroon	Prospective observational	Adult patients referred for upper gastrointestinal endoscopy from 2014 to 2015 to university teaching hospitals at Yaounde and Douala.	43.8 (20 to 71)	27/29	56	34	Endoscopic	ART Era
Rubaihayo et al. [18]	Uganda	Retrospective observational	Patients attending TASO HIV Clinic in 4 TASO centers in Uganda from 2002 to 2009	33 (27 to 40)	39379/69240	108619	8778	Non-endoscopic	ART Era
Brentlinger et al. [20]	Mozambique	Prospective observational	HIV-infected ambulatory patients 18 years of age and above presenting for scheduled (routine) or unscheduled care at three participating health centers, with current Hb < 10 g/dl in 2012	29 (23 to 36)	62/262	324	4	Non-endoscopic	ART Era
Bagny et al. [19]	Togo	Prospective observational	All adult HIV patients admitted to hepato-gastroenterology department of the Centre Hospitalier Universitaire Lome´ campus fom January 1 to December 31, 2011	38.8 (20 to 65)	15/67	82	24	Endoscopic	ART Era
Preidis et al. [21]	Malawi	Retrospective observational	Data from all HIV patient September 1, 2007, and December 31, 2008	21.8 (11.1 to 44.6)	479/405	884	35	Non-endoscopic	ART Era
Zachariah et al. [22]	Malawi	Retrospective cohort	HIV patients receiving care in Thylo District health centers from 2006 to 2008	35	706/1610	2289	53	Non-endoscopic	ART Era
Okeke et al. [26]*	Nigeria	Retrospective observational	Patients who underwent endoscopy at Jos University Teaching Hospital	–	−/−	52	18	Endoscopic	Pre-ART
Mohamed et al. [10]	Kenya	Prospective observational	Consecutive patients 12 years and above referred between 2000 and 2001	32 (18 to 55)	20/32	52	27	Endoscopic	Pre-ART
Morgan et al. [24]	Uganda	Prospective observational	Prevalent and incident community cases from 1989 to 1998	Males: 36.9 Females: 36.5	38/34	72	19	Non-endoscopic	Pre-ART
Ravera et al. [23]	Uganda	Randomized clinical trial	From September 1994 to December 1995, 320 consecutive AIDS patients were observed at the Gastroenterology Department of Hoima Hospital in Uganda	23.7	27/50	320	77	Endoscopic	Pre-ART
Kelly et al. [28]*	Zambia	Cross-sectional	HIV seropositive patients presenting with persistent diarrhea at the University Teaching Hospital, Lusaka	Male: 30 Female: 29.5 Range: 16–64	40/35	75	41	Endoscopic	Pre-ART

Abbreviations: *AIDS* acquired immunodeficiency syndrome, *ART* anti-retroviral therapy, *TASO* The AIDS Support Organization, *IQR* interquartile range, *M* male, *F* female, *HIV* human immunodeficiency virus

*Not included in the meta-analysis as the selection of the patient was from a restricted population with a higher likelihood of over estimation of the prevalence of OC

### Prevalence of esophageal candidiasis

**Fig. 2:**
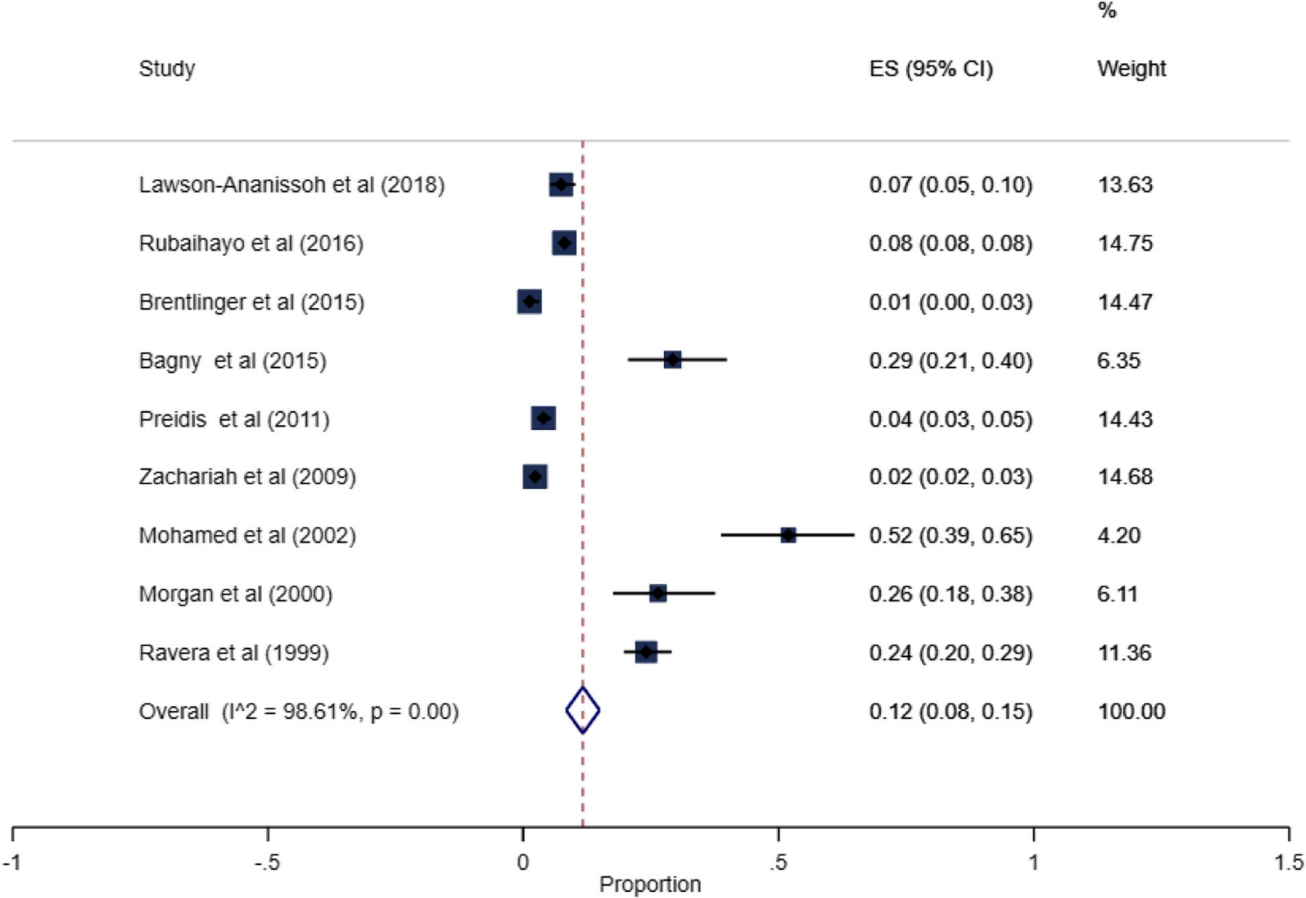
The pooled prevalence of HIV-associated esophageal candidiasis in sub-Saharan Africa

**Fig. 3:**
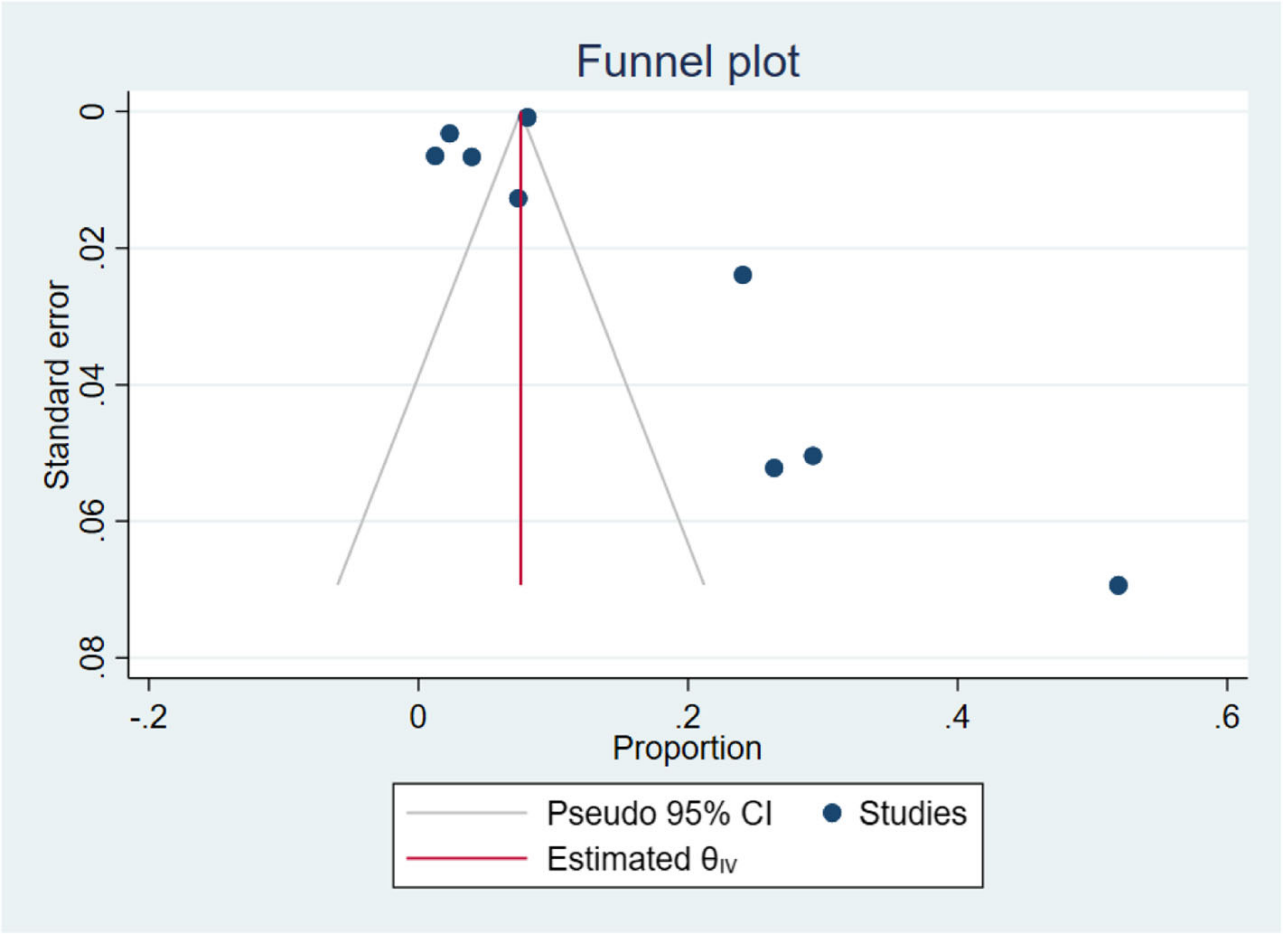
A funnel plot showing the distribution of eligible studies

Four of the 13 studies recruited a more restricted group of patients [25–28], with the prevalence of OC ranging between 28% and 55%. These studies were excluded from the meta-analysis. Of the 9 studies included in the meta-analysis, the pooled prevalence of HIV-associated OC in SSA was 12% (95% CI. 8 to 15%, *I*^2^ = 98.61%, *P* < 0.001), Fig. 2. The prevalence ranged from 1 to 52% across individual studies. Only two studies [17, 18] were within the funnel plot, Fig. 3. No study described the microbiological etiology of the *Candida* species responsible.

### Trends in HIV-associated esophageal candidiasis

**Fig. 4:**
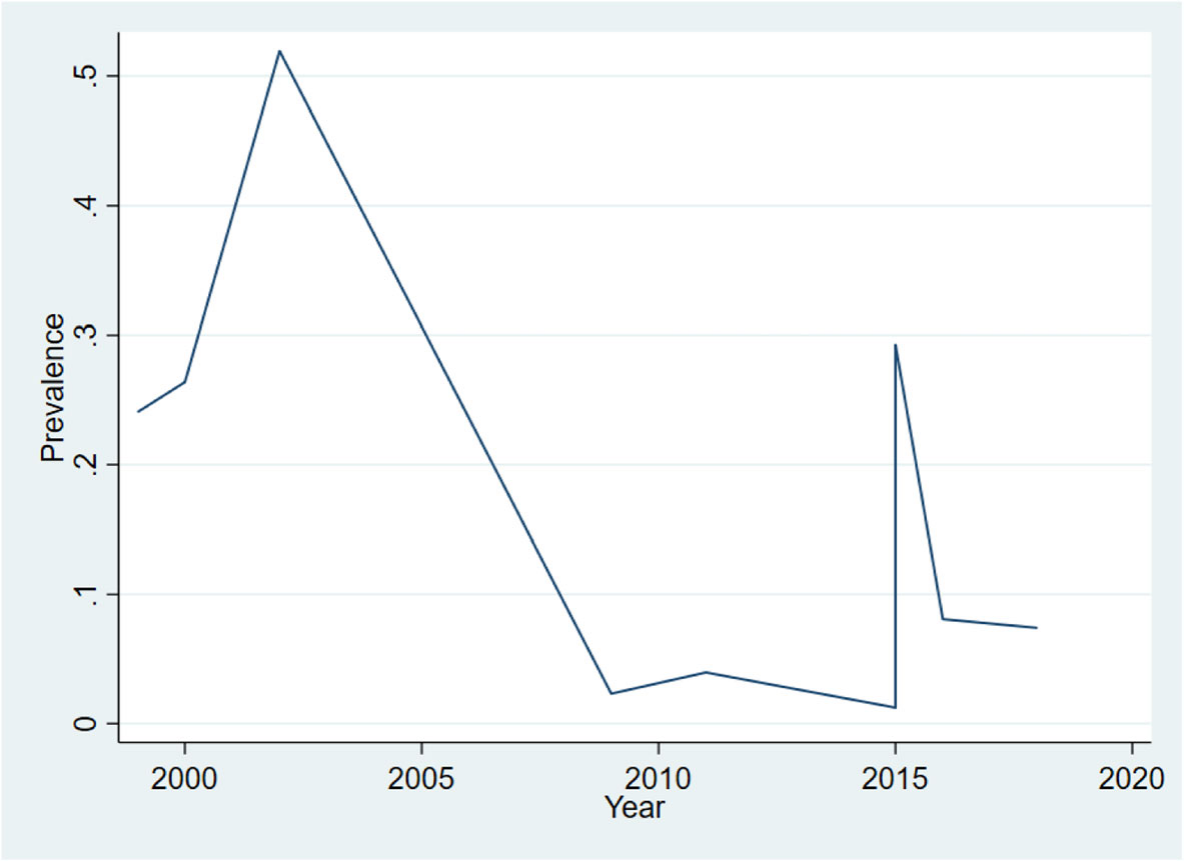
Trends in the prevalence of HIV-associated esophageal candidiasis in sub-Saharan Africa

**Fig. 5:**
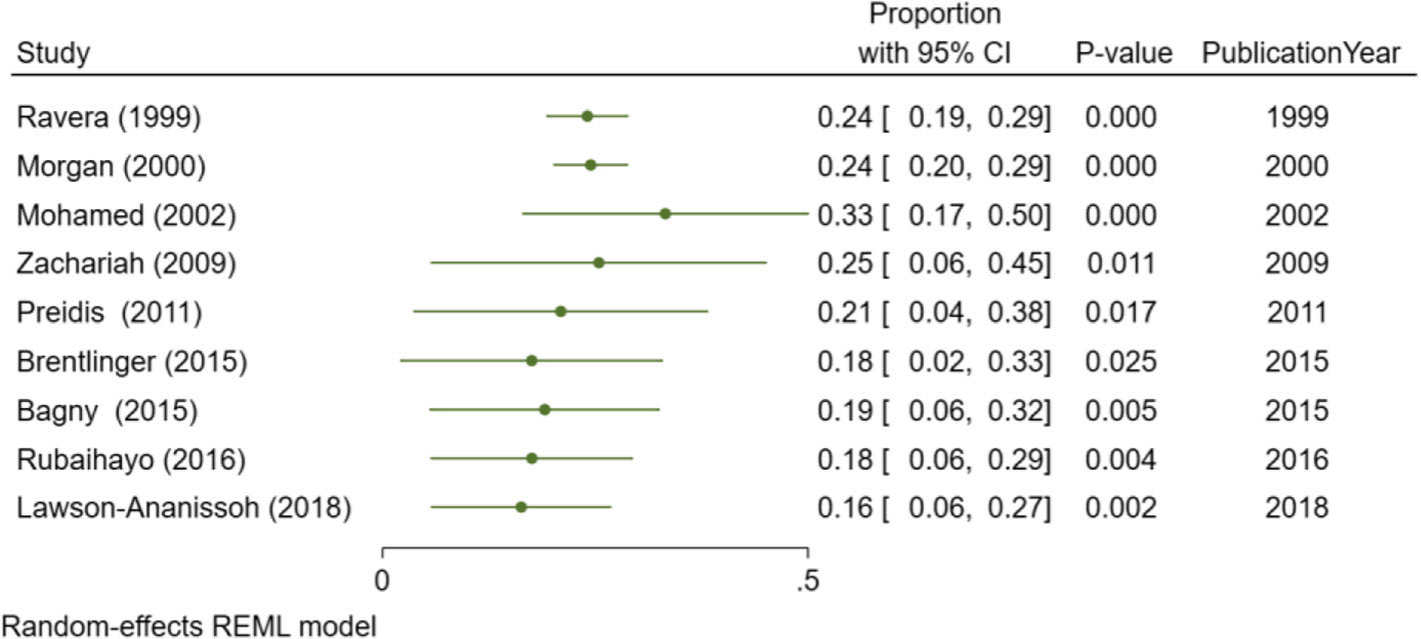
Cumulative prevalence of esophageal candidiasis in HIV patients for the period 1994 to 2014

Overall, the prevalence of OC significantly decreased over the over three decades of the HIV pandemic in SSA, Figs. 4 and 5. Cumulative prevalence ranged from 24% in 1999 to 16% in 2018, Fig. 5. OC was frequently reported with oral candidiasis in some studies [10, 23].

### Diagnosis of esophageal candidiasis

**Fig. 6:**
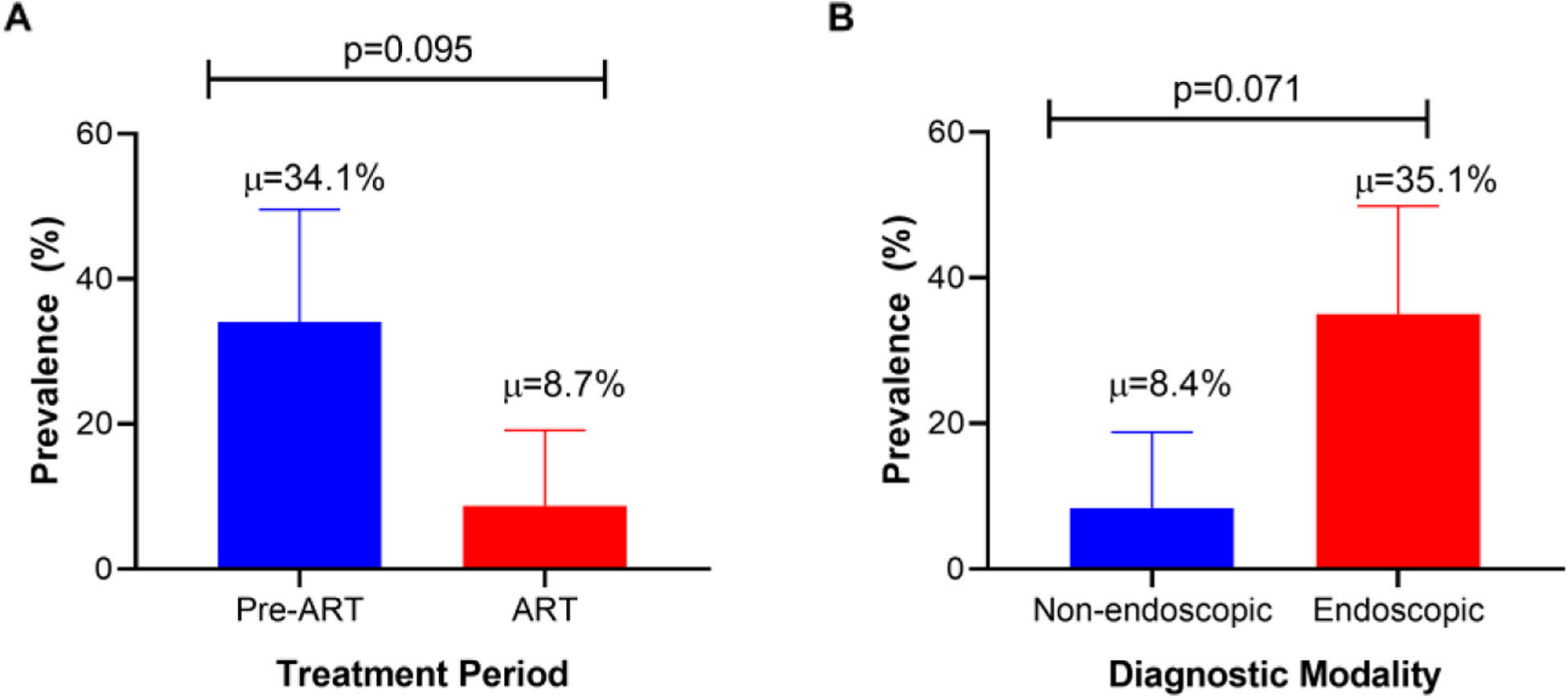
Mean prevalence of HIV-associated esophageal candidiasis by antiretroviral therapy era (**a**) and diagnostic modality (**b**)

In 7 studies, diagnosis of OC was solely made through upper gastrointestinal endoscopy, non-endoscopic (clinical or histology) in 5 studies, and not specified in one study (Table 1). Overall, the mean prevalence of OC in the 9 studies included in quantitative synthesis was higher in endoscopic diagnosis (35.1%) than in non-endoscopic diagnoses (8.4%), but this was not statistically significant (*p* = 0. 071), Fig. 6.

### Antiretroviral therapy and esophageal candidiasis

**Fig. 7:**
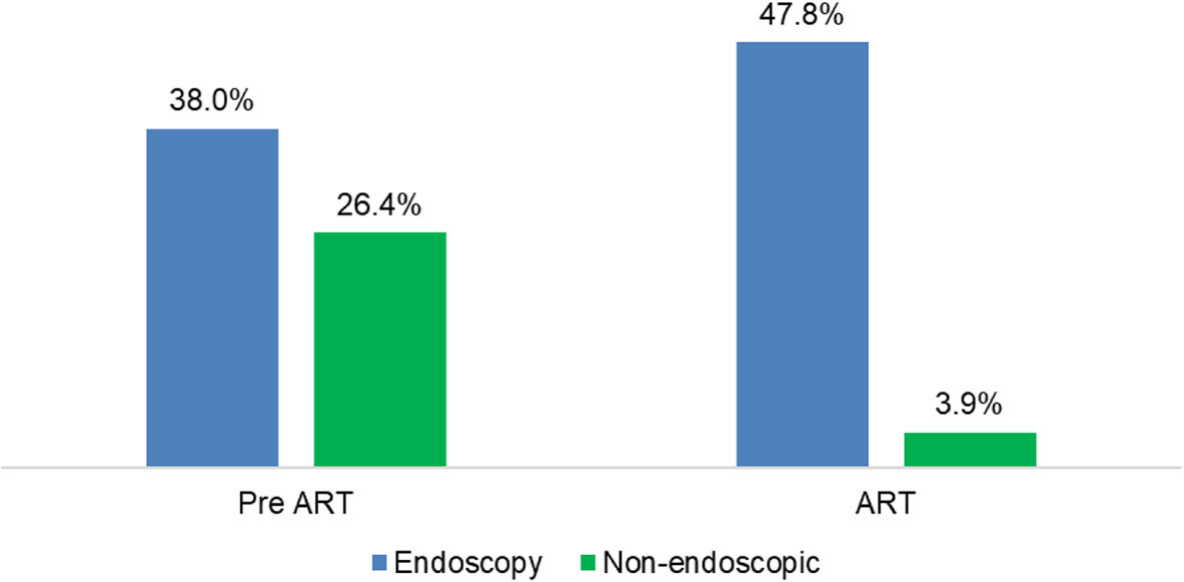
Mean prevalence of HIV-associated esophageal candidiasis in the pre-ART and ART era by diagnostic modality

Five studies were conducted during the pre-ART era and 8 studies during the ART era (Table 1). The mean prevalence of OC in the 9 studies included in quantitative synthesis was higher in studies conducted during the pre-ART era (34.1%) compared to the ART era (8.7%). However, this difference was not statistically significant (*p* = 0.095, Fig. 6). In sub-analysis, the mean prevalence of HIV-associated OC was higher in endoscopically diagnosed patients than in non-endoscopic diagnosed PLHIV during both pre-ART and ART era (17.8% vs. 4.6%). However, this difference was not statistically significant (*p* = 0.071), Fig. 7.

## Discussion

This systematic review aimed to determine the prevalence and the trend in the prevalence of OC in the pre-ART and ART era. Overall, in this large review, the pooled prevalence of OC was 12% (95% CI, 8 to 15%), which is comparable to the global data on the lifetime prevalence (10 to 15%) of OC among patients with HIV/AIDS [6]. A significant decline in the prevalence of OC was observed between 1996 and 2015 from as high as over 65% to as low as < 10%. This decline reflects the introduction and the widespread use of ART across SSA, mainly supported by the President’s Emergency Plan for AIDS Relief (PEPFAR) initiative launched in 2004. Consistent use of ART improves immunological response and leads to viral suppression in PLHIV with a subsequent decline in the frequencies of opportunistic infections, including OC [29–31]. The small increase in the prevalence of OC observed between 2012 and 2014 could be due to the increasing number of the “late presenters” [32, 33]. That is, patients presenting to care with advanced HIV disease or those already on ART re-presenting to care with advanced HIV disease due to treatment failure [34, 35]. The prevalence of OC was higher in those diagnosed endoscopically compared to those diagnosed clinically irrespective of the ART era. This could be due to the low sensitivity of clinical diagnosis for the definitive diagnosis of OC.

In a large study evaluating over 100,000 PLHIV in Uganda, the prevalence of OC was stratified into pre-ART period (male 9.8%, female 7.2%), early ART (male 10 %, female 7.2%) and late ART (male 2.9%, female 2.6%) [18]. However, the method of diagnosis of OC was not indicated in this study, but most likely clinical since a majority of these patients were managed as outpatients and esophagogastroduodenoscopy (OGD) is not widely available across the country.

In Malawi, OC significantly correlated with inpatient mortality (*p* < .05) with up to 8.8% of deaths reported [21]. In this study, the mean CD4 count of the patients who died with OC was 194.1 cells/mm^3^, and only 23.1% of the patients in the study were on ART. Mohamed and colleagues found that more esophageal lesions were significantly associated with CD4 counts less than 200 cells/mm^3^ [10]. Before the widespread use of ART, the median CD4 count for patients with OC was 101 (29 to 280) cells/mm^3^ in a Ugandan prospective study and the median survival time was 3.1 months in the untreated AIDS patients [24].

The diagnosis of OC remains a challenge in SSA. It is thus not surprising that in at least 4 of the studies we have evaluated, the diagnosis was made on a clinical basis [18, 20–22, 24]. OGD is not widely available, expensive for the patients and the expertise to perform the procedure is limited.

However, in most instances, suspected cases of OC are empirically treated with systemic antifungals. The resolution of classical symptoms of the disease (odynophagia and dysphagia) over several days of antifungal therapy is considered an alternative diagnostic approach [36]. Fluconazole and itraconazole are first-line agents that are equally efficacious. Alternative agents such as amphotericin B and the echinocandins are used in patients who have contraindications to, develop resistance, or are clinically failing while on systemic triazoles [36].

Lack of response to systemic antifungal response is uncommon and usually due to antifungal resistance or a wrong clinical diagnosis [36]. *C. albicans*, the most common cause of OC is usually susceptible to commonly prescribed triazole antifungals (fluconazole) [36]. However, in patients with previous exposure to the triazoles or those infected with non-*albicans Candida* species, treatment failure is common. *C. krusei* is intrinsically resistant to fluconazole but *C. dubliniensis* and *C. glabrata* which are more common in HIV have high minimum inhibitory concentrations to this commonly used antifungal [36]. Therefore, microbiological confirmation of the etiology and anti-fungal susceptibility testing is recommended for this group of patients [36]. In those clinically diagnosed, OGD should be performed to rule out infectious and malignant differential diagnoses such as herpes simplex virus (HSV), cytomegalovirus, and Kaposi’s sarcoma, which are common and may co-occur with OC [37].

### Limitations of the study

This study has some important limitations. Firstly, in over 10,000 cases of OC, the method of diagnosis was not specified or the disease was diagnosed clinically. This increases the risk of overestimation of the true burden of the disease; especially that HSV esophagitis and other viral esophagitis are equally common in the African population. Secondly, microbiological confirmation and speciation of the etiologic confirmation were not reported in all studies. These are very important areas for future studies. Thirdly, there was a very high degree of statistical heterogeneity in this study. This is due to the vast difference in the diagnostic methods, the population studied (denominator), and study design. However, we minimize this by performing sensitivity analysis and constructing a cumulative prevalence analysis in addition to the traditional pooled meta-analysis.

## Conclusion

Overall, our pooled data from this systematic review suggest a trend towards a declining prevalence of OC among PLHIV in the ART era in SSA. However, OC remains a common problem among PLHIV. Treatment outcomes and etiology of the disease largely remain unknown. This has a clinical implication in the choice of empirical antifungal therapy for suspected cases. Continuous active surveillance through OGD, empirical studies into microbiology and optimal antifungal treatment, and the impact of OC on quality of life of PLHIV in SSA are subjects for future studies.

## Data Availability

The datasets analyzed during the current study are available from the corresponding author on reasonable request.

## Abbreviations

OC: Esophageal candidiasis
SSA: Sub-Saharan Africa
ART: Antiretroviral therapy
HIV/AIDs: Human immune deficiency virus/acquired immune deficiency syndrome
PLHIV: Persons living with HIV
OGD: Esophagoduodenoscopy
